# Procedural sedation in the emergency department

**DOI:** 10.1186/cc12318

**Published:** 2013-03-19

**Authors:** MS Shah, FS Shah, KP Pope, AS Abbas

**Affiliations:** 1 Luton and Dunstable Hospital, London, UK; 2 Birmingham Hospitals NHS Trust, Birmingham, UK; 3 Chesterfield NHS Trust, Chesterfield, UK; 4 Colchester Hospital, Colchester, UK

## Introduction

Are safety guidelines being followed when administering procedural sedation in the emergency department? Between November 2004 and November 2008, the NPSA received 498 alerts of patients being given the wrong dose of midazolam for procedural sedation [1]. In the first 5 years of midazolam use there were 86 deaths, most related to procedural sedation [2].

## Methods

We searched through the controlled drugs book in resuscitation over a 2-month period and found a list of patients who had received midazolam or fentanyl. From this, we could make a search for the relevant A and E notes for these patients. From these notes, we looked for (see shorthand in Table 1): verbal consent documentation (consent), past medical history recorded (pmhx), safe initial dose of midazolam (midaz), pre-procedure monitoring (pre), post-procedure monitoring (post), and monitoring for 1 hour before discharge (1 hr). Following introduction of a reminder in the controlled drugs book/sedation room and staff education, the case notes were analysed over another 2-month period (24 sets of notes) to assess practise against safety guidelines.

## Results

See Table 1 (key for shorthand in Methods).

## Conclusion

The re-audit notices within the procedural sedation room and controlled drug book front cover served as a reminder of good practise. The visibility of this reminder (within the CD book) helped ensure better adherence to the audit standard. This reminder will now be kept within the CD book.

**Table 1:** Before and after education programme and reminder in the controlled drugs book

	Before (%)		After (%)	
		
	Yes	No	Yes	No
Consent	35	65	50	50
Pmhx	55	45	67	33
Midaz	58	42	88	12
Pre	25	75	54	46
Post	10	90	71	29
1 hr	55	45	54	46
